# Subtle Radiological Features of Splenic Avulsion following Abdominal Trauma

**DOI:** 10.1155/2010/762493

**Published:** 2010-12-01

**Authors:** S. A. Rehim, H. Dagash, P. P. Godbole, A. Raghavan, G. V. Murthi

**Affiliations:** ^1^Department of Paediatric Surgery, Sheffield Children's Hospital NHS Foundation Trust, Western Bank, Sheffield S10 2TH, UK; ^2^Department of Paediatric Radiology, Sheffield Children's Hospital NHS Foundation Trust, Sheffield S10 2TH, UK

## Abstract

Splenic trauma in children following blunt abdominal injury is usually treated by nonoperative management (NOM). Splenectomy following abdominal trauma is rare in children. NOM is successful as in the majority of instances the injury to the spleen is contained within its capsule or a localised haematoma. Rarely, the spleen may suffer from an avulsion injury that causes severe uncontrollable bleeding and necessitates an emergency laparotomy and splenectomy. We report two cases of children requiring splenectomy following severe blunt abdominal injury. In both instances emergency laparotomy was undertaken for uncontrollable bleeding despite resuscitation. The operating team was unaware of the precise source of bleeding preoperatively. Retrospective review of the computed tomography (CT) scans revealed subtle radiological features that indicate splenic avulsion. We wish to highlight these radiological features of splenic avulsion as they can help to focus management decisions regarding the need/timing for a laparotomy following blunt abdominal trauma in children.

## 1. Introduction

Nonoperative management of splenic trauma is well established in paediatric practice [1]. Patients who are haemodynamically stable can be safely treated with NOM [2]; it is associated with decreased incidence of postsplenectomy sepsis and complications associated with nontherapeutic laparotomies. A careful history, thorough clinical examination, and radiological investigations would guide overall management. However, management of children presenting with multiple injuries can often be challenging, and both clinical and radiological features may be subtle initially.

The aim of our report is to highlight some of these subtle radiological features associated with splenic avulsion injury, which may be difficult to interpret especially in the presence of multiple injuries.

## 2. Case I

An 11-year-old boy was admitted following a head-on collision with a stationary vehicle, after riding downhill on a bicycle without any headgear, approaching at a speed of around 25 miles/hr at moment of impact.

On arrival, his airway was patent and his cervical spine was immobilised in a collar. His pulse rate was 160/min, blood pressure 105/60 mmHg, respiratory rate 28/min, and oxygen saturation 99% on 15 L/min of oxygen. Glasgow Coma Scale at the scene was 4/15, improving to 8/15. Clinically evident injuries included a large degloving injury of the scalp, multiple facial and lip lacerations, comminuted fracture of the right humerus, and fracture of the left femur. He also had decreased air entry on the left side of the chest with some mild bruising on the left chest wall.

He was resuscitated with intravenous fluids and a urinary catheter was inserted, and this did not reveal any haematuria. Following elective intubation and ventilation, he underwent trauma series X-rays and contrast-enhanced computed tomography (CT) scans.

CT thorax revealed a substantial left-sided pneumothorax, which was relieved by a left chest drain. X-rays of his cervical spine and pelvis were normal. X-rays of his right humerus and femur revealed comminuted fractures of both long bones. CT head revealed normal appearances of the ventricles and basal cisterns with no intracranial haemorrhage; there was a small linear fracture of the left maxillary antrum.

**Figure 1:**
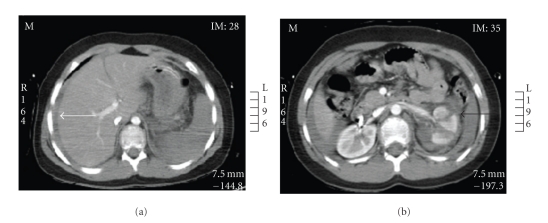
CT scan of the abdomen in portal venous phase showing fluid around the liver (white arrow), difficult to appreciate any splenic change. Lacerated left kidney with perinephric haematoma (black arrow).

Abdominal CT revealed a small amount of free fluid/blood seen around liver with multiple lacerations seen in the left kidney with a left perinephric and retroperitoneal haematoma. The left renal vein and artery appeared intact. There was no evidence of free intraperitoneal air or bowel wall thickening to suggest bowel trauma. The liver, spleen, pancreas, gall-bladder, and right kidney appeared normal; however, there was some image degradation artefact secondary to the arms being by the side of the abdomen (Figures 1(a) and 1(b)).

During CT scanning he became haemodynamically unstable and required further resuscitation with boluses of colloid and two units of blood. At this stage an emergent laparotomy was considered but not performed as he stabilised and was transferred to intensive care unit (ICU) for observation. His haemoglobin was 11.1 g/dL and platelet count 176. Urea and electrolytes were within the normal range.

Within the next 4 hours, following review by orthopaedic surgeons and neurosurgeons, he was taken to theatre for necessary operative interventions. These included suturing of lacerations, stabilisation of fractures, and insertion of an intracranial pressure (ICP) monitoring bolt. 

Over the subsequent 24 hours he remained haemodynamically unstable on the ICU with his haemoglobin dropping to 5.9 g/dL and a platelet count of 21. He received a further two units of blood plus cryoprecipitate. Examination revealed a soft abdomen with no evidence of bruising. Further 24 hours later he was hypotensive, urine output decreased, and he developed abdominal distension. Following a further blood transfusion, he underwent a repeat contrast-enhanced CT scan of his abdomen.

**Figure 2:**
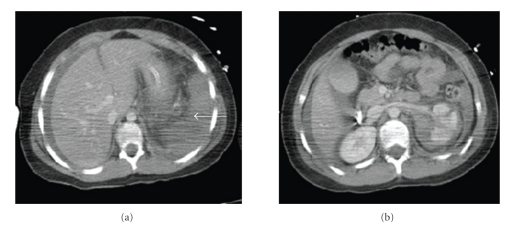
Followup CT scan of the abdomen in portal venous phase shows relative hypodensity of the spleen (white arrow), increase in intraperitoneal fluid, and unchanged appearance of  left kidney.

**Figure 3:**
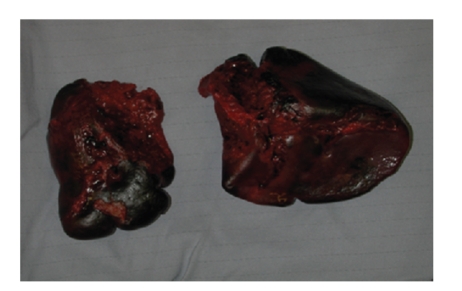
Case I: Appeared intact on CT but avulsed and in 2 pieces at laparotomy.

The CT scan revealed intraperitoneal blood, which had increased since the previous scan, mainly in the right and left paracolic gutter, lower abdomen, and pelvis. There was decreased enhancement of the spleen, suggesting a splenic injury (Figures 2(a) and 2(b)). He underwent an emergency laparotomy, and the spleen was found to be completely avulsed and in two pieces (Figure 3). The spleen was removed and the pedicle transfixed. He made an uneventful recovery and was discharged home on postoperative day 12.

## 3. Case II

A three-year-old boy was sitting on the handlebars of a motorcycle that was involved in a head-on collision with another motorcycle and suffered blunt trauma to his abdomen. He was admitted to the local hospitals A&E Department. Upon admission his pulse rate was 144/min, blood pressure 105/52 mmHg, and GCS 5/15. He was intubated ventilated, and subsequently trauma series X-rays and CT scans were performed.

Clinical findings of note were an abrasion to his lower anterior abdominal wall and bluish discoloration of his abdomen. The only positive radiological finding of note was the presence of free fluid in his abdomen on the abdominal CT scan. 

**Figure 4:**
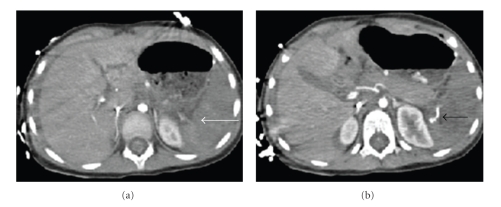
Contrast enhanced CT of the abdomen showing relative hypodensity of the spleen (white arrow), with extravasation of the contrast at the hilum suggestive of active bleeding (black arrow).

He underwent an emergency laparotomy because of persistent hypotension despite resuscitation. At laparotomy, his spleen was in three pieces and completely avulsed off its pedicle. There was a 30 cm long tear in the small bowel mesentery with the related gut showing signs of ischaemia. The rest of his bowel and solid organs were normal. He underwent splenectomy and resection of small bowel and end-to-end anastomosis. He made an uneventful recovery except for an incisional hernia that has been repaired successfully. Subsequent review of his CT scans revealed subtle features of nonenhancement of the spleen compared to the liver in the portal venous phase suggestive of splenic pedicle injury. There was active extravasation of contrast around the splenic hilum suggestive of ongoing active bleeding (Figures 4(a) and 4(b)).

## 4. Discussion

NOM of splenic trauma is well established in paediatric practice [1]. An avulsion injury to the spleen is rare and can occur following impact at great speed, where the spleen is torn off its pedicle. This would result in severe (case I) or uncontrollable (case II) haemorrhage and shock.

Splenic injury following blunt abdominal trauma is best diagnosed with the use of CT scan; intravenous contrast improves the sensitivity of this imaging modality [3]. In a nontrauma setting the spleen enhancement (56–65 HU) is slightly greater than that of the liver (40–60 HU). Berland and VanDyke [4] scanned ten random patients and found that splenic enhancement was on an average 23 Hounsfield units (HU) greater than hepatic enhancement. The splenic parenchyma reaches a homogeneous enhancement approximately 1 minute after contrast agent administration, with density values ranging between 75 and 97 HU.

Shock can lead to hypoperfusion of the spleen and result in poor enhancement on contrast-enhanced CT scans (Figure 2). However, following trauma, splenic enhancement may be decreased without any parenchymal or vascular injury [5], making interpretation of this subtle sign difficult. Furthermore, in an avulsion injury the solid organ may appear to be intact radiologically. In both cases above, the spleen appeared to be intact on both CT scans, and the difference in poorer enhancement of the spleen (30–45 HU) compared to the liver was subtle. (Figures 1 and 2).

In a state of hypovolemia, Detweiler [6] suggests the development of a hypoperfusion complex that affects the spleen more than the liver. Studies in canines demonstrate that stimulation of splenic nerve fibres caused decreased arterial inflow and increased venous outflow. This was associated with a decrease in splenic weight as blood was expelled from the spleen into the circulation. Anderson [5] postulated that a similar response might be observed in humans following trauma and hypotension. The reason the splenic perfusion is affected more than hepatic perfusion is possibly because hepatic arterial inflow is autoregulated and also because of the dual blood supply to the liver. Splenic enhancement by less than 30 HU in children (20 HU in adults) is a recognised feature of this hypoperfusion complex [4, 7]. Other features of vascular rupture or infarction of the spleen may be suspected with findings such as perisplenic haematoma, splenic enlargement, or rim enhancement.

Radiologists may also be able to comment on the patency of the hilar vessels and whether they are intact or not. The absence of adequate enhancement of the spleen in the portal phase would suggest a splenic vascular pedicle injury; the presence of extravasated contrast around the splenic hilum suggests active ongoing bleeding.

## 5. Conclusion

NOM remains the mainstay of managing splenic injury following blunt abdominal trauma. Radiological features of splenic avulsion can be subtle and difficult to interpret in the background of multiple injuries and persistent hypotension. In the context of blunt abdominal trauma, the density of the spleen on CT, as judged by Hounsfield units (HU), is an indicator of poor spleen perfusion. However, decision to go to laparotomy will continue to be based on clinical features of persistent shock despite resuscitation rather than specific identification of source of bleeding in the majority of cases. However, an awareness of the features highlighted above can help refine the decision making process, inform the surgeon regarding the operative approach and focus their search for source of bleeding during a difficult emergency laparotomy.

##  Conflict of Interests

This is no conflict of interests declared.
